# Determinants of Outcomes of Acute Kidney Injury: Clinical Predictors and Beyond

**DOI:** 10.3390/jcm10061175

**Published:** 2021-03-11

**Authors:** Emaad M. Abdel-Rahman, Faruk Turgut, Jitendra K. Gautam, Samir C. Gautam

**Affiliations:** 1 Division of Nephrology, University of Virginia, Charlottesville, VA 22908, USA; jkg8h@virginia.edu; 2 Internal Medicine/Nephrology, Faculty of Medicine, Mustafa Kemal University, Antakya/Hatay 31100, Turkey; turgutfaruk@yahoo.com; 3 Johns Hopkins School of Medicine, Baltimore, MD 21218, USA; sgautam6@jhmi.edu

**Keywords:** acute kidney injury, recovery, mortality, biomarkers

## Abstract

Acute kidney injury (AKI) is a common clinical syndrome characterized by rapid impairment of kidney function. The incidence of AKI and its severe form AKI requiring dialysis (AKI-D) has been increasing over the years. AKI etiology may be multifactorial and is substantially associated with increased morbidity and mortality. The outcome of AKI-D can vary from partial or complete recovery to transitioning to chronic kidney disease, end stage kidney disease, or even death. Predicting outcomes of patients with AKI is crucial as it may allow clinicians to guide policy regarding adequate management of this problem and offer the best long-term options to their patients in advance. In this manuscript, we will review the current evidence regarding the determinants of AKI outcomes, focusing on AKI-D.

## 1. Introduction

Acute kidney injury (AKI) is a clinical syndrome characterized by rapid (within hours to days) impairment of kidney function. Impaired kidney function during AKI leads to clinical consequences, including accumulation of nitrogen degradation products, fluid, acid-base and electrolyte imbalance, as well as non-renal organ dysfunctions and immune system impairment.

The Kidney Disease: Improving Global Outcomes (KDIGO) guidelines define AKI as an abrupt decrease in kidney function that occurs over a period of 7 days or less. The term acute kidney disease, which is a state between 7 and 90 days of AKI [1], has been proposed to define the course of disease after AKI among patients in whom the renal pathophysiologic processes are still ongoing.

AKI spans a broad spectrum of severity; AKI KDIGO classifications 1, 2, and 3 not requiring kidney replacement therapy (KRT), and AKI requiring dialysis (AKI-D) [1]. AKI-D is the most severe form of AKI and is associated with increased in-hospital mortality [2,3]. The prevalence of AKI-D was reported to be 1%–2% in hospitalized patients and 5%–13% in critically ill patients [3,4,5]. Unfortunately, AKI-D is on the rise, with its incidence increasing by 10% over a decade between 2000 and 2009 [6]. The ultimate goal for the care of patients with AKI-D is to achieve recovery. There is currently no standardized definition of kidney recovery in patients with AKI-D, but a consensus report of the Acute Disease Quality Initiative (ADQI) defined kidney recovery in these patients as sustained (> 14 days) independence from KRT [2].

Inpatient AKI may resolve spontaneously without needing KRT, require temporary inpatient dialysis, be discharged from the hospital while still requiring dialysis (AKI-D), or lead to death in the hospital. On the other hand, outpatient AKI may recover (partial or complete) or continue to have long-term renal complications as continuing to require dialysis, which could be for a prolonged period of time [7], progress to chronic kidney disease (CKD), end stage kidney diseases (ESKD), or death [4,5,8]. Furthermore, AKI is not only a localized disease to the kidneys, but may potentially cause remote effects, which may be associated with multiple consequences; increased cardiac events (congestive heart failure, coronary artery disease and stroke), recurrent hospitalization, and catheter-related infections [9,10].

Consequently, the high global burden of AKI, especially AKI-D, and the need for evidence-based clinical guidance make it vital to identify predictors of AKI-D outcome to guide further policy and further clinical care [11]. Predicting outcomes of these patients may allow clinicians to specify therapeutic needs and offer the different long-term options to their patients in advance. If a patient is predicted to remain dialysis-dependent, then subsequent care should be planned; education about KRT options, referral for a vascular access versus a peritoneal catheter and referral for renal transplantation evaluation. On the other hand, if a patient is more likely to recover and become dialysis-independent, measures to ensure avoiding renal insults need to be implemented, including avoiding hypotensive episodes on dialysis, avoiding nephrotoxic drugs, close monitoring of residual renal function, nephrology outpatient follow up once off dialysis, etc. Unfortunately, most existing studies examining predictors of outcomes after AKI-D have many important limitations.

In this manuscript, we will review the current evidence regarding the determinants of AKI-D outcomes. We will focus on several potential determinants of outcomes following AKI-D, including patients ‘demographics and co-morbidities, laboratory parameters, medications use, outpatient care of these patients, and biomarkers (Table 1). Our ultimate goal is to advise nephrologists about the potential reversible predictors of outcomes to help improve the outcomes of these patients (e.g., improve specific laboratory parameters, optimize dialysis prescription and adequately manage reversible comorbidities).

## 2. Predictors of Renal Outcomes

### 2.1. Demographics and Co-Morbidities

#### 2.1.1. Patient Demographics

A recent study that evaluated a cohort of more than a million incident dialysis patients over 10 years (1/1/2005–12/31/2014) using the US Renal Data System (USRDS), of whom 1.1% were on dialysis secondary to AKI (*n* = 11,498), showed that women, non-whites (Blacks, Asians, Hispanics and Native Americans), and elderly patients are less likely to achieve kidney recovery compared to men, whites and young patients, respectively [12]. Similarly, Foley and colleagues showed a lower recovery rate of kidney function in women, Blacks, and Hispanics compared to men, Whites, and non-Hispanics, respectively [13]. On the other hand, the effect of gender on mortality in patients with AKI-D was demonstrated in a study analyzing the French database (2009–2014, *n* = 64,870), showing that in-hospital mortality was significantly higher in males versus females (*p* < 0.001) [14]. Kohle et al., confirmed this finding of worse mortality in males following AKI [15]. The effect of age on renal recovery in patients with AKI-D was recently studied (*n* = 2214), showing that younger age is a strong predictor for kidney recovery [16]. Similar findings were noted indicating an increased mortality risk of elderly with AKI-D. A small retrospective study of elderly patients > 70 years with AKI-D (*n* = 137) showed that elderly patients with AKI-D had a very high rate of hospital mortality and among initial survivors within the first year after hospital discharge [17]. Garnier and colleagues confirmed the increased risk of in-hospital mortality among elderly French population with AKI-D [14]. Similar results were shown among patients with AKI in the United Kingdom [15]. A recent meta-analysis of 11 studies (*n* = 16,948) in patients with AKI on continuous kidney replacement therapy (CKRT) showed a correlation between increasing age and mortality with an odds ratio (OR) 1.023 (CI 1.006–1.04) [18].

Thus, compelling evidence from recent observational studies indicates that the elderly population seems to have worse outcomes after an episode of AKI-D, while the gender effect showed females to exhibit worse recovery and better survival after the AKI-D episode compared to males.

#### 2.1.2. Co-Morbidities

##### Severity of Acute Disease

The effects of the severity of acute disease and high illness severity (Charlson comorbidity index and APACHE II score) associated with AKI have been assessed in sveral studies and showed that the less severity of the acute diseases was a predictor for kidney recovery and less mortality after the onset of AKI-D [15,18,19,20,21,22].

##### Pre-Existing CKD

Several studies noted that surviving patients with normal baseline kidney function eventually recover after an AKI-D episode [5,23,24], suggesting that baseline estimated glomerular filtration rate (eGFR) may be an important determinant of AKI-D outcomes. A recent study showed that patients with eGFR ≥ 30 mL/min/1.73 m^2^ had a significantly higher likelihood of kidney function recovery, even after adjustment for intensive care unit (ICU) initiation, catheter access, sepsis/postoperative etiology of AKI, acute tubular necrosis and heart failure (HR, 5.86, *p* < 0.001) [21]. Researchers at the university of Virginia evaluated outcomes of patients with AKI-D following their hospital discharge, and showed that patients with baseline eGFR <45 mL/min are 88% less likely to achieve dialysis-independence, both short- term [7] and long-term [25]. Similar studies further highlighted the effect of baseline renal function on kidney function recovery [21,24,26].

##### Prior AKI

AKI is associated with short- and long-term sequelae. In patients surviving after an AKI-D episode, AKI may be an important determinant of future AKI risk. Gautam and colleagues showed that patients with prior AKI had odds of non-recovery of a new episode of AKI-D that is almost 75% greater than those without a history of prior AKI [7]. Furthermore, in a retrospective study evaluating patients with new AKI (*n* = 359), recurrent AKI occurred in 34% of their population. In these patients with recurrent AKI episodes, CKD, cardiovascular events and mortality were significantly higher than those with no recurrent AKI (*p* = 0.003, 0.001 and < 0.001, respectively) [27].

##### Diabetes Mellitus

Diabetes Mellitus is not only a major cause of CKD, but it is also an independent risk factor for the development of AKI [28]. Patients with diabetes with either preserved kidney function or CKD at baseline are more likely to experience AKI and recurrent AKI compared with non-diabetic CKD controls [28]. In a recent study, Harding and colleagues noted that the presence of diabetes in patients with AKI-D was a predictor of a higher rate of hospitalization; almost 5 times as high in adults with diabetes compared with adults without diabetes [29].

##### Cardio-Vascular Diseases

AKI frequently occurs in patients with cardiovascular diseases; chronic hypertension and heart failure are not only risk factors for AKI but may also negatively affect recovery from AKI [30]. We further showed that patients with AKI-D who had CHF were 69% less likely to achieve dialysis independence [7]. Lindner and colleagues showed similar results [31]. These deleterious effects of CHF on kidney recovery of AKI-D patients persist even in patients with higher baseline eGFR [21].

##### Volume Overload

Several studies from both adult and pediatric literature highlighted the grave consequences of fluid overload on patients with AKI initiating dialysis [32,33]. Outcomes of AKI patients with fluid overload include increased mortality, decreased kidney recovery, increased hospital length of stay (LOS) and longer need for mechanical ventilation [34,35]. Chua and colleagues showed that a cumulative median of fluid balance of more than 8 L in patients with AKI-D correlated with increased hospital mortality compared to less than 8 L at the initiation of dialysis [36]. The grave effect of volume overload in patients with AKI extended along with different clinical scenarios; post-operative cardiac surgery, general critical setting and in patients on extracorporeal membrane oxygenation (ECMO) [35,37,38].

##### COVID-19

The new challenge that the world is recently witnessing with the COVID-19 pandemic makes studies aiming at developing a tool to predict outcomes of AKI even more compelling. COVID-19 is a disease caused by SARS-CoV-2, a novel virus that has caused a pandemic with staggering rates of mortality and morbidity world-wide. COVID-19 virus affects many organs, including the kidneys. It has been reported that this virus is responsible for variety of renal manifestations, the most common being proteinuria and hematuria [39].

AKI has also been reported to complicate the course of the COVID- 19 virus, more so, after pulmonary involvement [40]. One third of hospitalized patients with COVID- 19 infection have been shown to develop AKI but the rates are variable based on geography.

Compared to non-COVID- 19 hospitalized patients, the COVID-19 patients have higher rate of AKI across all the stage of AKI with more than double the rate of AKI-D as shown in a recent multicenter study [41]. Among these patients, the duration of dialysis was longer in patients infected with COVID- 19 compared to non-COVID 19 hospitalized patients. The mortality rate in patients with AKI following COVID- 19 infection was higher compared to the patients without this infection (29.6% vs. 11.3%, *p* < 0.001) [41].

The causes of AKI are varied according to the clinical stage of COVID-19 infection. Within the first weeks of COVID-19 infection, the most common cause of AKI is intravascular volume depletion due to poor oral intake, vomiting and prostration. However, in the later stage of the COVID infections, AKI can occur mainly as a consequence of acute tubular injury due to hemodynamic insult, sepsis, mechanical ventilation, vasopressor use or direct viral infection of the tubules. The most common kidney biopsy finding in patients with AKI following COVID-19 viral infection was acute tubular injury [42]. Other causes of AKI following COVID-19 infection, are collapsing glomerulopathy and thrombotic micro-angiopathies [43].

Managing COVID-19- associated AKI-D has been challenging because of scarcity of resources from dialysis fluids to human manpower. Acute peritoneal dialysis has been successfully used in these patients. Other challenges noted in the management of these patients were coagulation and disposition [44]. As these patients were hypercoagulable, frequent clotting in CRRT was noted even while on anticoagulation [44]. From disposition standpoint, as recovery of renal function in these patients was less than in the non- COVID patients, finding appropriate disposition in long term facilities and dialysis units has been difficult.

##### Other Co-Morbidities

AKI is common in hospitalized patients with chronic liver disease who had reduced chances for kidney recovery due to compromised kidney perfusion [45]. Similarly, patients with chronic health conditions, including cancer and chronic obstructive pulmonary disease have increased risk of AKI and worse outcomes [46,47,48,49]. A recent study reported that patients with AKI-D were not associated with better survival in patients with stage IV cancer [50]. AKI, especially AKI-D, is common and is associated with serious complications after both cardiovascular and non-cardiac surgery [51]. Patients who require dialysis after surgery may have higher rates of mortality and kidney non-recovery [52,53].

In summary, baseline renal function, prior AKI, volume overload and other non-renal co-morbid diseases can modify the risk of renal recovery and both short- and long-term mortality. Among them, volume overload is the only potentially modifiable factor. Better management strategies of chronic disease may be helpful to decrease the risk of AKI-D.

### 2.2. Laboratory Parameters

Several studies attempted to develop applicable tools using laboratory parameters to predict the probability of kidney recovery and survival among patients with AKI. Despite that, no tool was validated as an AKI-D outcome prediction model. While a model consisting of four variables, including pre-admission hemoglobin level was reported to have a moderate ability to identify the probability of kidney recovery in patients with AKI [16], high pre-AKI-D level of proteinuria and increased serum creatinine level at CKRT initiation were demonstrated to be an independent risk factor for non-recovery of patients with AKI-D [18].

To evaluate the ability of several laboratory parameters to predict mortality in patients with AKI, Saly and colleagues used stepwise regression to analyze data from a randomized AKI trial [54]. They showed that increased serum anion gap, hypomagnesemia and hyperkalemia correlated with more death in these patients after initiation of dialysis in the hospital. Increased serum bicarbonate positively correlated with longer hospital LOS in the same study [54].

In patients undergoing cardiac surgery, pre-operative albuminuria was shown not only to be independently associated with AKI, but also independently associated with AKI-D and mortality [55].

A recent Korean study examined patients undergoing CKRT for AKI in an intensive care setting (*n* = 1558) and suggested that high creatinine: cystatin C ratios were associated with better survival [56]. Furthermore, Gao and colleagues showed that among AKI patients in ICU (*n* = 13,621), hypo- and hypernatremia, hypo- and hyperkalemia were associated with increased mortality [57]. The combination of hyponatremia and hyperkalemia was associated with the worst mortality [57]. Kolhe et al. analyzed data from the United Kingdom Intensive Care National Audit and Research Center Case Mix Programme aiming to evaluate the outcomes of patients with severe AKI (*n* = 17,326) during the first 24 h of ICU admission. They noted that increased mortality among these patients correlated with hypo- and hypernatremia, hyperkalemia (but not hypokalemia), severe hypoalbuminemia (<2.0 g/L), leukopenia and leukocytosis [15].

Based on the available data, several laboratory data findings may affect and predict outcomes of AKI. Further studies are needed to further evaluate the use of some of these laboratory parameters as viable models and markers to predict outcomes of patients with AKI.

### 2.3. Medications

#### 2.3.1. Renin Angiotensin Inhibitors (RASi)

New start of RASi or continuing the use of these agents versus holding them for patients with or at risk for AKI has been controversial. While some studies showed some benefit with the use of these agents with improved outcomes of AKI, other studies failed to show any improvement [58,59]. In a retrospective study comparing mortality among veteran patients with AKI resuming (*n* = 19,968) versus not resuming (*n* = 10,205) angiotensin receptor blockers (ARBs) post non-cardiac surgery, the authors noted worsening 30 days mortality in the group withholding ARB (3.2% vs. 1.3%). Similar results were noted when using ACE inhibitors (ACEi) [58,59], suggesting a beneficial role for resuming RASi in patients who suffered an episode of AKI.

Brar et al. recently reported on adults who experienced an episode of AKI during hospitalization (*n* = 46,253) [60]. Receipt of RASi therapy within 6 months after hospital discharge was associated with reduced mortality with no difference in ESKD. This beneficial effect of RASi on mortality in patients with AKI was confirmed in another study [61]. In addition to the beneficial role of using RASi on mortality in patients with AKI, Chou et al. reported that among patients who had cardiac surgery–associated AKI (*n*= 587), the use of RASi was associated with a lower risk of ensuing CKD [62]. 

On the other hand, Hines and colleagues showed that using RASi before or during an episode of AKI did not affect kidney recovery [63]. Hsu and colleagues recently studied a cohort of RASi-naïve patients with AKI post-hospitalization (*n* = 10,242). They noted no statistical difference in recurrent hospitalized AKI between patients who were newly started on RASi versus those not receiving RASi (5.7% vs. 6.1%) [64]. Similarly, Bidulka and colleagues recently showed no increased risk of heart failure or subsequent AKI in patients continuing RASi after an episode of AKI compared to those stopping these agents [65].

Taken together, these studies suggest that the use of RASi can have a potential benefit and may be able to predict a better outcome (i.e., better survival and improved kidney recovery).

#### 2.3.2. Diuretics

Ejaz and Mohandas reviewed literature assessing the role of diuretics in decreasing the transition of AKI to CKD. They noted that while diuretics can help with volume management in these patients with AKI, diuretics play no role in the management of AKI [66].

More recently, Zhao and colleagues analyzed data of critically ill patients with AKI (*n* = 14,154) and compared outcomes (LOS, recovery of renal function and in-hospital and 90-day mortality) based on whether they received furosemide or not. They noted that the group with KDIGO AKI stages 2–3, based on urine output criteria, that received furosemide had a reduced in-hospital and 90-day mortality (*p* < 0.001), and improved recovery of kidney function (*p* < 0.001) [67]. Other studies agreed with these findings even in patients with AKI-D. Katayama and colleagues studied patients with AKI-D and showed that positive urinary response to a dose of diuretics was a good predictor of recovery from CKRT [68]. Other studies confirmed the positive role of the urine output in predicting dialysis independence following AKI-D [18,69,70].

These results suggest a role of diuretics in improving outcomes following AKI but need further confirmations.

#### 2.3.3. Pressors

Controversial results were published when assessing the role of pressors on the outcomes of AKI. The differences noted in the results could be related to the type of pressor used and the clinical scenario in which the pressor was used. In a multi-center observational study, Chen and colleagues assessed patients with septic AKI-D (*n* = 372) to evaluate the impact of nor-epinephrine on 90-day mortality and kidney recovery. They showed that norepinephrine was associated with a significantly higher 90-day mortality rate signifying a strong predictor of the 90-day mortality (*p* < 0.03) [71]. This outcome was worse compared to other vasoactive agents and that the detrimental effect of norepinephrine was dose-dependent. Several other studies agreed with these results [72,73].

Other studies using different pressors showed different outcomes. Pajewski and colleagues showed that the use of vasopressors was associated with better kidney recovery [70]. Tumlin and colleagues evaluated the impact of angiotensin II on the outcomes of AKI-D in patients with catecholamine resistant vasodilatory shock (CRVS)**.** They showed a superior impact of angiotensin II on survival through 28 days and renal recovery within 7 days compared to placebo [74]. Bellomo and colleagues further showed that the elevated serum renin concentration in these patients with CRVS might identify patients who will benefit the most with using angiotensin II [75].

### 2.4. Outpatient Care of Patients with AKI-D

#### 2.4.1. Dialysis-Related Factors

##### Renal Replacement Modality

Studies examining the outcome of AKI-D showed different outcomes based on the dialysis modality used. CKRT is considered by some to be superior to intermittent hemodialysis (IHD) in managing AKI-D [3,76,77,78,79], as this modality causes less intradialytic hypotension and may provide better control of fluid balance and more hemodynamic stabilization [80]. Conger and Schrier were among the earliest scientist to study the association between the changes in systemic and renal hemodynamics and AKI. They discussed several pathogenetic theories that may potentially link hemodynamic changes and AKI. These theories included renin-angiotensin system stimulation, redistribution of renal blood flow and intrarenal coagulation [81]. Schneider and colleagues evaluated 23 studies of patients with AKI-D, including both randomized controlled trials (RCTs) (7 studies) and observational studies (16 studies). They noted that while RCTs failed to show any difference in outcomes related to the initial choice of KRT modality, the observational studies suggested a lower rate of renal recovery among patients starting on IHD initially compared to those starting initially on CKRT [79].

Kellum and colleagues showed better kidney recovery with the use of CKRT compared to IHD. This advantage noted by CKRT was not maintained when multivariate analysis was done [69]. Pajewski and colleagues supported the findings that a higher proportion of patients on CKRT had a better chance of kidney recovery (71.4% vs. 31.4%) in comparison to IHD [70]. On the other hand, other studies failed to show a significant difference in AKI outcome among different dialysis modalities on kidney recovery [23,80,82,83,84,85,86,87].

The role of peritoneal dialysis (PD) in managing AKI-D cannot be overlooked, more so now during the COVID-19 pandemic. PD may provide hemodynamic stability, preserve residual renal function, with less inflammatory cascade and fewer blood stream infections [88]. PD was shown to be associated with better kidney recovery of AKI-D and better survival [89,90]. These findings contradicted with other studies. A Cochrane meta-analysis of 6 studies found no significant differences in death or kidney recovery between patients treated with PD versus other extracorporeal therapies [91]. More data are needed to further define the outcome of acute PD in these patients.

##### Dose and Duration of RRT

The timing of initiating dialysis seems to not affect outcomes in patients with AKI [23,85,86]. On the other hand, the intensity of KRT and prolonged duration of dialysis-dependence in these patients may be associated with poor outcomes. In a post hoc analysis of the Acute Renal Failure Trial Network (ATN) study, increasing the intensity of KRT was associated with less likelihood for kidney recovery at 28 days in patients with AKI [92]. Rathore and colleagues showed minimal renal recovery of patients with AKI-D beyond 90 days of outpatient dialysis [22].

##### Intradialytic Hypotensive Episodes

Frequent hypotensive episodes with 3 or more hypotensive episodes during dialysis were associated with renal non-recovery in patients with AKI-D (24.6%) vs. only one hypotensive episode (9.3%) with no difference in the dialysis duration [70]. This finding suggests the need for close follow up and adopting practice guidelines aiming at adjusting antihypertensive medications at frequent intervals, avoiding aggressive ultrafiltration with frequent adjustments of the target weight and the use of diuretics to off load the volume.

##### Biocompatible Membranes

Effects of dialysis filter membrane biocompatibility on renal recovery in patients with AKI-D are not well defined. Hakim and colleagues prospectively studied patients with AKI-D (*n* = 72) randomized to 2 different dialysis filter membranes. They showed better renal recovery (62% vs. 37%) and improved patients’ survival (57% vs. 37%) in the group of patients using biocompatible dialysis filter membrane vs. those using bio-incompatible dialysis filter membranes [93]. This result was confirmed by others [94]. Alonso and colleagues disagreed with these results. They reviewed 10 studies (*n* = 1100) and found no clinical advantages on using biocompatible membrane vs. bio-incompatible membranes [95].

#### 2.4.2. Nephrology Follow Up Post AKI

Follow up by nephrologist post AKI-D recovery has shown to predict better outcomes. Wu and colleagues studied patients with AKI-D (*n* = 20,250) who became dialysis-independent. They compared outcomes of these patients when followed by a nephrologist (*n* = 7550) versus not (*n* = 12,700) [96]. They demonstrated better survival and fewer major adverse cardiovascular events (MACE) in the group, followed by a nephrologist. Ikizler and colleagues further showed that in patients with AKI (*n* = 769), the majority of whom had AKI stage 1, the deleterious effects of AKI on survival and heart failure were attenuated when patients were followed and assessed for kidney function recovery and proteinuria three months after AKI [97].

### 2.5. Biomarkers

While several biomarkers were identified that may be able to predict AKI episodes, very few data are available identifying biomarkers predicting outcomes following an AKI episode. Currently, potential biomarkers predicting AKI include functional biomarkers as a rise in serum creatinine along with urine output. Though serum creatinine is not a perfect marker in patients with chronic illnesses and prolonged hospitalizations with loss of muscle mass, they are still extensively used as kidney function markers. A rise in Cystatin-C (Cyst-C) level was also shown to be predictive of kidney function. Cyst-C is produced by nucleated cells, freely filtered, reabsorbed, and metabolized in the kidney. Serum Cyst-C is proposed to be a better kidney function marker than serum creatinine in a certain population [98].

Recently, many kidney damage-associated biomarkers that may be able to predict an AKI episode have been extensively reviewed, including Chitinase 3-like protein 1 (CHI3L1), Neutrophil gelatinase-associated lipocalin (NGAL), Kidney injury molecule-1, Liver fatty acid binding protein (L-FABP), osteopontin, hemojuvelin and interleukins [99,100,101,102,103,104].

More recently, two new biomarkers were identified as predictors of AKI; insulin-like growth factor binding protein 7 (IGFBP-7) and the tissue inhibitor of metalloproteinases-2 (TIMP-2) [105]. IGFBP-7 is a member of insulin growth factor binding proteins with a molecular mass of 30 kDa. It binds and inhibits signaling through IGF-1 receptors. Urinary IGFBP-7 is increased in kidney damage caused by sepsis or ischemic insult. TIMP-2 is a 21 kDa protein, a member of the tissue inhibitor of matrix metalloproteinase family. TIMP2 is an endogenous inhibitor of metalloproteinase activities and participates in the regulation of cell growth and apoptosis. Its downstream signaling results in arrest of endothelial cell proliferation via activation of kinases. Both IGFBP-7 and TIMP-2 are stress markers leading to temporary cell cycle arrest as an immediate response to kidney injury to prohibit maladaptive repair. Cell cycle arrest for a longer duration leads to increased chances of fibrosis and CKD.

The combination of IGFBP-7 and TIMP-2 was found to be superior in predicting AKI compared to previously available biomarkers. The Opal and Topaz trials established the AKI risk assessment cut-off value for urinary [TIMP-2] x [IGFBP-7] [106,107]. The test (NephroCheck) is FDA approved and is commercially available for AKI risk prediction within the United States [108]. AKI is strongly predicted with NephroCheck cut off > 0.3 and a value > 2.0 indicates AKI incidence within 12–24 h. Studies suggested that these markers may help in predicting kidney recovery [109,110]. The role of TIMP-2 and IGFBP7 in predicting post AKI recovery or progression to ESKD or death is proposed but has not been adequately studied.

The recently published Ruby study highlights a new biomarker, C-C motif chemokine ligand 14 (CCL-14) [111]. The study was prospective and observational that analyzed data from 391 ICU patients with KDIGO stage 2 or 3 AKI and demonstrated that elevated urinary CCL-14 level was a predictor of non-renal recovery and persistent stage 3 AKI [103]. CCL14 is a member of the chemokine family of small molecules recognized for monocyte/macrophage recruitment and is associated with pro-inflammatory chemotaxis. It is implicated in tissue injury and repair processes. A higher urinary level of CCL-14 implicates an inflammatory state with higher pro-inflammatory immune cell recruitment and polarization. This resulting chronic inflammatory state can promote tissue damage.

The RUBY study also tested previously known AKI markers for their ability to predict AKI persistence. Other biomarkers that came close but did not cross 0.75 area under the curve (AUC) threshold for AKI persistence were: urine CHI3L1, plasma cystine-C, urine NGAL, plasma proenkephalin, urine L-FABP and Nephrocheck biomarkers (TIMP-2 and IGFBP7).

Another recently published study pointed to the role of NGAL in predicting persistent AKI. In this retrospective study (*n* = 1322), the authors showed that the urine NGAL/Creatinine ratio was a better predictor of persistent AKI versus transient AKI (104).

A panel of kidney injury markers was recently tested as predictors of AKI recovery. Of all the markers tested for the prediction of recovery, a combination of urinary Ganglioside M2 activator protein (GM2AP) and tail-less complex polypeptide-1 eta subunit (TCP1-eta) predicted recovery with a success rate of around 80% [112]. GM2AP and TCP1-eta50 are urinary biomarkers for tubular and cortical tubular damage, respectively. Neither TIMP-2, IGFBP-7, nor CCL-14 were tested in this study [105].

In summary, while there is currently no single methodology that predicts AKI outcomes, several promising approaches are being studied. Knowing the importance of predicting outcomes of AKI, an interdisciplinary, well-established, collaborative approach is needed to improve the care and experiences of AKI patients. Further studies are needed that combine clinical data, laboratory data, medications used, and dialysis parameters, along with biomarkers to establish the best methodology to predict outcomes of AKI.

While some of the predictors of AKI outcomes are non-modifiable, others are modifiable. Modifying these factors may potentially have a positive impact on AKI outcomes, which in turn, may have a huge positive impact on patients’ survival and their quality of life in addition to a positive economic impact by avoiding unnecessary procedures.

## Figures and Tables

**Table 1:** Determinants of AKI-D outcomes.

Determinants of AKI-D Outcomes
• Demographics and Co-morbidities
• Patient demographics
• Co-morbidities
• Severity of acute disease
• Pre-existing CKD
• Prior AKI
• Diabetes Mellitus
• Cardio-vascular diseases
• Volume overload
• Other co-morbidities: Chronic liver diseases, cancer, surgeries.
• Laboratory parameters
• Medications
• Renin Angiotensin Inhibitors (RASi):
• Diuretics
• Pressors
• Outpatient care of Patients with AKI-D
• Dialysis-Related Factors
• Renal Replacement Modality
• Dose and Duration of RRT
• Intradialytic Hypotensive Episodes
• Nephrology follow up post AKI
• Biomarkers
• uCCL-14
• uNGAL/Creatinine ratio
• uGM2AP
• uTCP1-eta

Abbreviations: uCCL-14: urinary C-C motif chemokine ligand 14, uNGAL: urinary Neutrophil gelatinase-associated lipocalin, uGM2AP: urinary Ganglioside M2 activator protein, uTCP1-eta: tail-less complex polypeptide-1 eta subunit.
